# Die bauliche Struktur der deutschen Intensivstationen aus dem Blickwinkel der Infektionsprävention: Eine Umfrage unter den ITS-KISS-Teilnehmern

**DOI:** 10.1007/s00063-023-01022-x

**Published:** 2023-06-06

**Authors:** Giovanni-Battista Fucini, Christine Geffers, Frank Schwab, Michael Behnke, Julia Moellmann, Wolfgang Sunder, Petra Gastmeier

**Affiliations:** 1 Institut für Hygiene und Umweltmedizin, Charité – Universitätsmedizin Berlin, Hindenburgdamm 27, 12203 Berlin, Deutschland; 2 Nationales Referenzzentrum für Krankenhausinfektionen (NRZ), Hindenburgdamm 27, 12203 Berlin, Deutschland; 3 IKE Institut für Konstruktives Entwerfen, Industrie- und Gesundheitsbau, Technische Universität Carolo Wilhelmina zu Braunschweig, Pockelsstr. 3, 38106 Braunschweig, Deutschland

**Keywords:** Infektion, Einzelzimmer, Hygiene, Architektur, Umfrage, Cross-infection, Single-bed room, Hygiene, Architecture, Survey

## Abstract

**Einleitung:**

Die bauliche Struktur der Intensivstation (ITS) kann eine wichtige Rolle in der Infektionsprävention spielen.

**Methoden:**

Wir haben im Zeitraum von 09/2021 bis 11/2021 eine Umfrage zur aktuellen baulichen Struktur von ITS im deutschsprachigen Raum durchgeführt.

**Ergebnisse:**

Insgesamt 597 (40 %) Stationen haben geantwortet. 20 % davon wurden vor 1990 gebaut. Die mediane Anzahl der Einzelzimmer inklusive „interquartile range“ (IQR) beträgt 4 (IQR 2–6), die mediane totale Zimmeranzahl ist 8 (IQR 6–12). Die mediane Zimmergröße beträgt 19 m^2^ (IQR 16–22) für Einzelzimmer und 31 m^2^ (IQR 26–37,5) für Mehrbettzimmer. 80% der ITS haben ein Waschbecken und 86,4 % eine raumlufttechnische Anlage im Patientenzimmer. 54,6 % der Stationen müssen Geräte und Materialien außerhalb von Lagerräumen lagern und nur 33,5 % haben einen Raum für die Aufbereitung von Medizinprodukten. Aus der Stratifizierung nach Baujahr hat sich eine langsame Zunahme in der Ausstattung mit Einzelzimmern (3 [IQR 2–5] vor 1990 vs. 5 [IQR 2–8] nach 2011; *p* < 0,001) in den letzten 30 Jahren gezeigt.

**Diskussion:**

Die Ausstattung der ITS mit Einzelzimmern und deren Größe entspricht noch nicht den Forderungen der nationalen Fachgesellschaften. Auf vielen Stationen mangelt es an Platz für die Lagerung und für die Aufbereitungsprozesse von Medizinprodukten.

**Fazit:**

Es gibt einen dringlichen Bedarf, den Neubau und die Sanierung von ITS in Deutschland mit adäquaten Mitteln zu fördern.

## Abstract (en)

**Introduction:**

Intensive care unit (ICU) structural and spatial design may play a role in infection prevention and control.

**Methods:**

Between 09/2021 and 11/2021 we performed an online survey among ICUs in Germany, Austria and Switzerland.

**Results:**

A total of 597 (40%) of the invited ICUs answered the survey; 20% of the ICUs were built before 1990. The median number of single rooms with interquartile range is 4 (IQR 2–6). The median total room number is 8 (IQR 6–12). The median room size is 19 (IQR 16–22) m^2^ for single rooms and 31 (26–37.5) m^2^ for multiple bed rooms. Furthermore, 80% of ICUs have sinks and 86.4% have heating, ventilation, air conditioning (HVAC) systems in patient rooms. 54.6% of ICUs must store materials outside of storage rooms due to lack of space and only 33.5% have a room dedicated to disinfection and cleaning of used medical devices. Comparing ICUs built before 1990 and after 2011 we could show a slightly increase of single rooms (3 [IQR 2–5] before 1990 vs. 5 [IQR 2–8] after 2011; *p* < 0.001).

**Discussion:**

A large proportion of German ICUs do not meet the requirements of German professional societies regarding the number of single rooms and size of the patient rooms. Many ICUs lack storage space and other functional rooms.

**Conclusion:**

There is an urgent need to support the construction and renovation of intensive care units in Germany with adequate funding.

Einige bauliche Aspekte im Gesundheitswesen können eine relevante Rolle in der Infektionsprävention haben. Vor allem in Bereichen mit stark infektionsgefährdeten Patient*Innen ist eine gut geplante Umgebung sehr wichtig, um infektionspräventive Maßnahmen konsequent implementieren zu können. Dieser Beitrag will die aktuelle bauliche Struktur von Intensivstationen (ITS) in Deutschland und im deutschsprachigen Raum untersuchen, um kritische Punkte und Herausforderungen für die Zukunft zu identifizieren.

## Einleitung

Nach den Daten der letzten nationalen Prävalenzstudie 2016 haben am Untersuchungstag ca. 17 % der Patienten auf ITS in Deutschland eine nosokomial erworbene Infektion [5]. Nosokomiale Infektionen können schwerwiegende Konsequenzen für die Patienten haben, außerdem stellen sie eine erhebliche Belastung für das Gesundheitssystem dar, führen zu längerer Liegedauer und erhöhten Aufenthaltskosten [4, 6].

Die bauliche Struktur der ITS kann eine Rolle in der Infektionsprävention spielen. Die ausschließliche Unterbringung in Einzelzimmern könnte die nosokomiale Infektionsrate und die Erwerbsrate von multiresistenten Erregern senken [15, 16, 21]. Eine verbesserte Lokalisation der Händedesinfektionsmittelspender kann die Compliance für die Händedesinfektion erhöhen [22]. Die Entfernung von Wasserquellen aus dem Patientenzimmer [13] und die Optimierung der Raumbelüftung [18] wären effektiv, um die Kontamination der Umgebung und die Übertragung von Krankheitserregern zu reduzieren. Stiller et al. haben im Jahr 2015 bereits eine Umfrage zur baulichen Struktur der deutschen ITS durchgeführt. Größere ITS (> 12 Betten) und ITS in größeren Krankenhäusern (> 319 Betten) hatten höheren Kolonisationsraten mit multiresistenten Erregern und insgesamt höhere Infektionsraten. Darüber hinaus schien die Möglichkeit der Fensterlüftung im Patientenzimmer protektiv gegen nosokomiale Pneumonien zu wirken [20].

Die Deutsche Interdisziplinäre Vereinigung für Intensivmedizin (DIVI) hat im Jahr 2010 und dann im Jahr 2022 konkrete Empfehlungen für die Ausstattung von ITS herausgegeben. In der neuesten Version wurden einige Anforderungen z. B. bezüglich Anteil und Größe der Patientenzimmer weiter erhöht [23].

Die COVID-19-Pandemie hat außerdem eindeutig gezeigt, dass ungünstige bauliche Verhältnisse – z. B. wegen eingeschränkter Isolationsmöglichkeiten – zu Problemen in der Infektionsprävention führen können. Mit der vorliegenden Studie wird der aktuelle, detaillierte Stand der baulichen Struktur der ITS in Deutschland – v. a. in Hinblick auf die Möglichkeiten der Infektionsprävention – erfasst.

Eine Bestandaufnahme von vorhandenen Strukturen kann Bund und Länder helfen, bei der Finanzierung der Krankenhausplanung die Schwerpunkte für notwendige Investitionen zu identifizieren.

## Materialien und Methoden

Das Krankenhaus-Infektions-Surveillance-System (KISS) erfasst Daten über nosokomiale Infektionen und multiresistente Erreger aus freiwillig teilnehmenden Krankenhäusern in Deutschland, Österreich und der Schweiz seit 1997. Die 1500 Stationen, die mindestens einmal in den vorherigen 18 Monate Daten an das KISS gesandt haben, wurden in der Umfrage eingeschlossen.

Die Mitarbeiter, die im Nationalen Referenzzentrum für Surveillance von nosokomialen Infektionen (NRZ) zuständig für die Infektionserfassung hinterlegt sind (meistens Hygienefachkräfte), wurden am 06.09.2021 per E‑Mail zur Teilnahme an der Umfrage eingeladen und gebeten, den Fragebogen in Zusammenarbeit mit den Stationsleitungen auszufüllen. Personen, die für die KISS-Erfassung auf mehreren Stationen zuständig waren, haben für jede Station einen separaten Fragebogen bekommen. Für die Onlinebefragung wurde LimeSurvey© (2006–2021 LimeSurvey GmbH, Hamburg, Deutschland) genutzt. Die zu der Umfrage benutzten Server befinden sich im Rechenzentrum der Charité. Die Dauer der Umfrage betrug 10 Wochen.

Zu den abgefragten Aspekten zählten unter anderem das Baujahr und die Zugangswege der ITS, die Anzahl der Funktionsräume (Pflegestützpunkt, Lagerräume etc.), die Isolationsmöglichkeiten, die Anzahl und Größe von Patientenzimmern und die Voraussetzungen für die Aufbereitung von Betten und den Einsatz der Medizinprodukte.

Für die Beschreibung der Einzel- und Mehrbettzimmer wurden die Teilnehmer gebeten, die tatsächlichen Daten von 2 „typischen“ Zimmern der Station als Beispiel zu wählen.

In einer Subgruppenanalyse für die deutschen ITS wurden die Antworten mit den folgenden Grundcharakteristika der Stationen stratifiziert:

- Baujahr (vor 1990, zwischen 1991 und 2000, zwischen 2001 und 2010, ab 2011);
- Träger (privat vs. nichtprivat);
- Fachrichtung (interdisziplinär im Haus mit < 400 Betten, interdisziplinär im Haus mit ≥ 400 Betten, internistisch, chirurgisch, neurochirurgisch, kardiochirurgisch, neurologisch, pädiatrisch, Weaning, sonstige);
- Bettenanzahl der ITS (ITS mit < 12 Betten vs. ITS mit ≥ 12 Betten);
- Versorgungslevel des Krankenhauses (Grund- und Regelversorgung vs. Schwerpunkt und Maximalversorgung);
- Bettenanzahl im ganzen Krankenhaus (< 400 Betten vs. ≥ 400 Betten).

Die erhobenen Daten wurden deskriptiv ausgewertet und nachfolgend entsprechend in Anzahl und Anteil in (%) sowie als Median und „interquartile range“ [IQR] präsentiert. Unterschiede wurden mittels χ^2^-Testung oder Wilcoxon-Rangsummentest auf Signifikanz geprüft. Signifikant war ein *p*-Wert < 0,05.

Die statistische Auswertung erfolgte mit SPSS (Version 26) und SAS (Version 9.4).

## Ergebnisse

Von den 1500 angeschriebenen ITS-KISS-Teilnehmern aus Deutschland, Österreich und der Schweiz haben 597 (39,8 %) an der Umfrage teilgenommen. Die teilnehmenden Stationen repräsentieren 2523 Einzel-, 2736 Mehrbettzimmer und insgesamt 7115 Intensivbetten. Die Grundcharakteristika der teilnehmenden ITS sind in Tab. 1 dargestellt.

**Table 1:** 

Grundcharakteristika der Stationen		N	%
Gesamt		597	–
Fachrichtung	Interdisziplinär	354	59
	Internistisch	71	12
	Chirurgisch	74	12
	Kardiochirurgisch	19	3
	Neurochirurgisch	13	2
	Pädiatrisch	15	2
	Kardiologisch	11	2
	Neurologisch	11	2
	Traumatologisch	4	1
	Brandverletzte	8	1
	Sonstiges	17	3
Anzahl Betten	≥ 12	341	57
	< 12	256	43
Art des Krankenhauses	Regelversorgung	202	34
	Schwerpunktversorgung	126	21
	Maximalversorgung – Universitätsklinikum	99	17
	Maximalversorgung – andere	68	11
	Grundversorgung	66	11
	Fachkrankenhaus	29	5
	Reha-Einrichtung	4	1
	Sonstige	3	0,5
Art des Krankenhausträgers	Öffentlich	288	48
	Kirchlich	141	24
	Privat	104	17
	Freigemeinnützig	47	8
	Sonstige	17	3
Land	Deutschland	542	91
	Österreich	53	9
	Schweiz	2	0,3
Baujahr	Vor 1990	142	24
	1991–2000	195	32
	2001–2010	142	24
	Ab 2011	118	20

^a^NRZ-Definitionen: Grundversorgung: weniger als 5 Abteilungen, Bettenzahl > 20 bis < 200; Regelversorgung: 5 bis 10 Abteilungen, Bettenzahl > 200 bis < 800; Schwerpunktversorgung: mehr als 10 Abteilungen, Bettenzahl > 800; Fachkrankenhaus: nur spezielle Abteilungen. Definition von Krankenhausträger und Fachrichtung gehen an die jeweiligen Stationen zurück. Die Fachrichtung richtet sich nicht nach der leitenden Abteilung, sondern nach der Art der Patienten, die vorwiegend behandelt werden

Tab. 2 beschreibt die Zugangswege zur Station und Tab. 3 die Charakteristika der wichtigsten Funktionsräume (Stützpunkt, Lagerraum, reine und unreine Arbeitsräume).

**Table 2:** 

Frage	Ja (*n*)	Ja (%)
*Gibt es auf der Intensivstation eine Schleuse für die Einschleusung des Personals?*	267	44,7
Ist in der Schleuse mindestens ein Handdesinfektionsmittelspender vorhanden?	265	99,3
Wird die Schleuse auch als Umkleide benutzt?	232	86,9
*Gibt es auf der Intensivstation eine Schleuse ausschließlich für die Einschleusung von Besuchenden?*	185	31
Ist in der Schleuse mindestens ein Handdesinfektionsmittelspender vorhanden?	184	99,5
Können die Besuchenden in der Schleuse privates Eigentum ablegen?	144	77,8
Können die Besuchenden in der Schleuse ggf. Schutzkleidung anziehen?	153	82,7
*Gibt es auf der Intensivstation eine Schleuse ausschließlich für die Versorgung bzw. Entsorgung von Materialien?*	249	41,7
Werden saubere Materialien als auch kontaminierte Materialien über dieselbe Schleuse ein- und ausgeschleust?	34	13,7
Wenn ja: Wird darauf geachtet, dass saubere und kontaminierte Materialien sich nicht gleichzeitig in der Schleuse befinden – z. B. Lieferung und Abholung zum unterschiedlichen Zeitpunkt im Tagesverlauf	31	91,2
*Gibt es auf Ihrer Intensivstation einen Raum für die Aufnahme und Übergabe von Patienten, die aus anderen Bereichen bzw. anderen Krankenhäusern kommen?*	103	17,3
Hat dieser Raum einen Vorraum?	14	13,6

**Table 3:** 

Frage	Ja (*n*)	Ja (%)	Median (IQR)
*Gibt es auf Ihrer Intensivstation Patiententoiletten außerhalb der Patientenzimmer (z.* *B. auf dem Flur), die von mehreren Patienten bzw. für deren Versorgung benutzt werden können?*	306	51,3	–
*Wie viel Platz (Gesamtfläche) ist als Lagerraum auf Ihrer Station deklariert (Angabe in m*^*2*^*)?*	–	–	40 (24;60)
*Werden Geräte/Materialien ausschließlich in dafür deklarierten Räumen gelagert?*	271	45,4	–
Wenn nein: Erfolgt das aus Platzmangel in den Lagerräumen?	276	46,2	–
*Wie viele Pflegestützpunkte gibt es auf Ihrer Intensivstation?*	–	–	1 (1;1)
*Befindet sich der Pflegestützpunkt in einem räumlich abgeschlossenen Bereich?*	261	44	–
Verfügt der Pflegestützpunkt über die Möglichkeit der direkten Fensterlüftung?	81	31	–
*Findet in dem Pflegestützpunkt eine Lüftung über eine raumlufttechnische (RLT-)Anlage statt?*	477	80,2	–
*Wie viele Arbeitsplätze sind in dem Pflegestützpunkt vorhanden?*	–	–	3 (2;4)
*Gibt es auf Ihrer Intensivstation einen oder mehrere reine Arbeitsräume außerhalb der Patientenzimmer für die Vorbereitung von Infusionen oder für andere aseptische Tätigkeiten?*	466	78,1	–
Wie viele?	–	–	1 (1;1)
Gibt es eine feste Zuordnung zwischen Patientenzimmern und reinen Arbeitsräumen?	185	39,7	–
*Gibt es auf Ihrer Intensivstation einen oder mehrere unreine Arbeitsräume außerhalb der Patientenzimmer? (Unreine Arbeitsräume in Vorraum/Schleuse vor Patientenzimmer sind hier ausgenommen.)*	570	95,5	–
Wie viele?	–	–	2 (1;2)
Gibt es eine feste Zuordnung zwischen Patientenzimmern und unreinen Arbeitsräumen?	249	43,7	–

*IQR* „interquartile range“

576 Stationen (96,5 %) haben einen Aufenthaltsraum für das Personal im Stationsbereich mit einer Mediangröße von 20 (IQR 15;26) m^2^. Die Hälfte der teilnehmenden ITS (306, 51,3 %) hat ein Patientenbad, das vom Flur aus von mehreren Patienten benutzt werden kann.

Die Tab. 4 und 5 beschreiben die Isolationsmöglichkeiten (Vorräume, Isolierzimmer) und die relevanten Charakteristika der Patientenzimmer.

**Table 4:** 

Frage	Ja (*n*)	Ja (%)	Median (IQR)
*Wie viele Vorräume sind insgesamt auf Ihrer Intensivstation vorhanden?*	–	–	2 (1;3)
*Verfügen eines oder mehrere Einbettzimmer über einen eigenen Vorraum?*	386	64,7	–
Wie viele?	–	–	2 (1;3)
Sind die Vorräume mit Unterdruckoption ausgestattet?	214	55,4	–
Wie viele?	–	–	1 (0;2)
Verfügen die Vorräume über einen unreinen Arbeitsbereich mit Steckbeckenspüle?	250	64,8	–
Wie viele?	–	–	1 (0;2)
Ist in diesen genug Abstand zwischen dem reinen und dem unreinen Bereich vorhanden?	193	77,2	–

*IQR* „interquartile range“

**Table 5:** 

Frage	Ja (*n*)	Ja (%)	Median (IQR)
*Über wie viele Einbettzimmer verfügt Ihre Intensivstation?*	–	–	4 (2;6)
Wie groß ist das Zimmer? (Angaben in m^2^)	–	–	19 (16;22)
Befindet sich ein patientenbezogener Computer im Patientenzimmer?	261	48,6	–
Wenn ja: Befindet sich dieser Computer direkt am Patientenbett?	194	74,3	–
Sind im Patientenzimmer eine oder mehrere Waschbecken vorhanden?	430	80,1	–
Wird das Wasser aus dieser Wasserleitung für die Patientenversorgung genutzt?	360	83,7	–
Ist das Waschbecken mit Filtern ausgestattet?	219	50,9	–
Wird in diesem Waschbecken auch unreines Wasser entsorgt?	189	44	–
Befindet sich ein in Armlänge erreichbarer Handdesinfektionsmittelspender am Patientenbett?	405	75,4	–
*Über wie viele 2‑ oder Mehrbettzimmer (>* *2‑Betten) verfügt Ihre Intensivstation?*	–	–	4 (2;6)
Wie groß ist das Zimmer (Angaben in m^2^)?	–	–	31 (26;38)
Wie groß ist der Abstand (Seite zu Seite) zwischen den Betten von (Angaben in cm)?	–	–	200 (150;200)
*Findet in den Patientenzimmern eine Lüftung über eine raumlufttechnische (RLT-)Anlage statt?*	516	86,4	–
*Kann in mindestens ein Patientenzimmer durch die Lüftungsanlage ein Über bzw. Unterdruck zur Schleuse/Flur generiert werden?*	330	55,3	–

*IQR* „interquartile range“

49,9 % der Stationen bereiten benutze Patientenbetten auf der Station auf. Die Aufbereitung von Geräten und Patientenbetten ist in Tab. 6 dargestellt.

**Table 6:** 

Frage		Ja (*n*)	Ja (%)
Findet die Aufbereitung von benutzten Patientenbetten auf der Station statt?	–	298	49,9
Wo findet die Aufbereitung von benutzten Patientenbetten statt?	Im Patientenzimmer	180	60,4
	Im Flur	88	29,5
	In einem speziell dafür vorgesehenen Raum	30	10,1
Ist ein vorgesehener Abstellplatz für jeweils saubere und unsaubere Betten auf der ITS oder in unmittelbarer Nähe vorhanden?	–	187	62,5
Findet die Aufbereitung von folgenden Geräten nach Benutzung auf der Station statt?	Spritzenpumpen	587	98,3
	Beatmungsgeräte (mobil)	573	96
	Sonographiegeräte	550	92,1
	Mobile Monitoreinheiten	533	89,3
	Beatmungsgeräte (Patientenzimmer)	523	87,6
	Dialysegeräte	384	64,3
	Laryngoskope	176	29,5
	TEE-Sonden	164	27,5
	NO-Inhalationsgeräte	158	26,5
	ECMO-Maschinen	118	19,8
	Bronchoskope	32	5,4
Gibt es auf Ihrer Station einen Extraraum für die Aufbereitung von Geräten nach Benutzung?	–	200	33,5
Wird UVC-Strahlung zur Desinfektion der Luft oder von Oberflächen eingesetzt?	–	15	2,5
Werden festinstallierte oder mobile UVC-Desinfektionsgeräte angewendet?	Festinstallierte	0	0
	Mobile	15	100

*ITS* Intensivstation, *ECMO* Extra-corporal membran-Oxygenierung, *TEE* Transesophageale Echokardiographie, *NO* Nitrous Oxid (Stickstoffmonoxid)

Aus der Subgruppenanalyse der Stationen aus Deutschland (*n* = 542) geht hervor, dass der Anteil der Stationen, die in den letzten 5 Jahren umfassend renoviert worden sind, sich nicht signifikant nach dem Baujahr unterscheidet (Tab. 7).

**Table 7:** 

	Ab 2011	2001–2010	1990–2000	Vor 1990	*p*-Wert
Anzahl Stationen nach Baujahr (*n*, %)	121 (22,3 %)	180 (33,2 %)	132 (24,4 %)	109 (20,1 %)	–
Anteil davon, die in den letzten 5 Jahren umfassend renoviert wurden (*n*, %)	39 (32,2 %)	41 (22,8 %)	48 (36,4 %)	33 (30,3 %)	0,062

**Figure 1:**
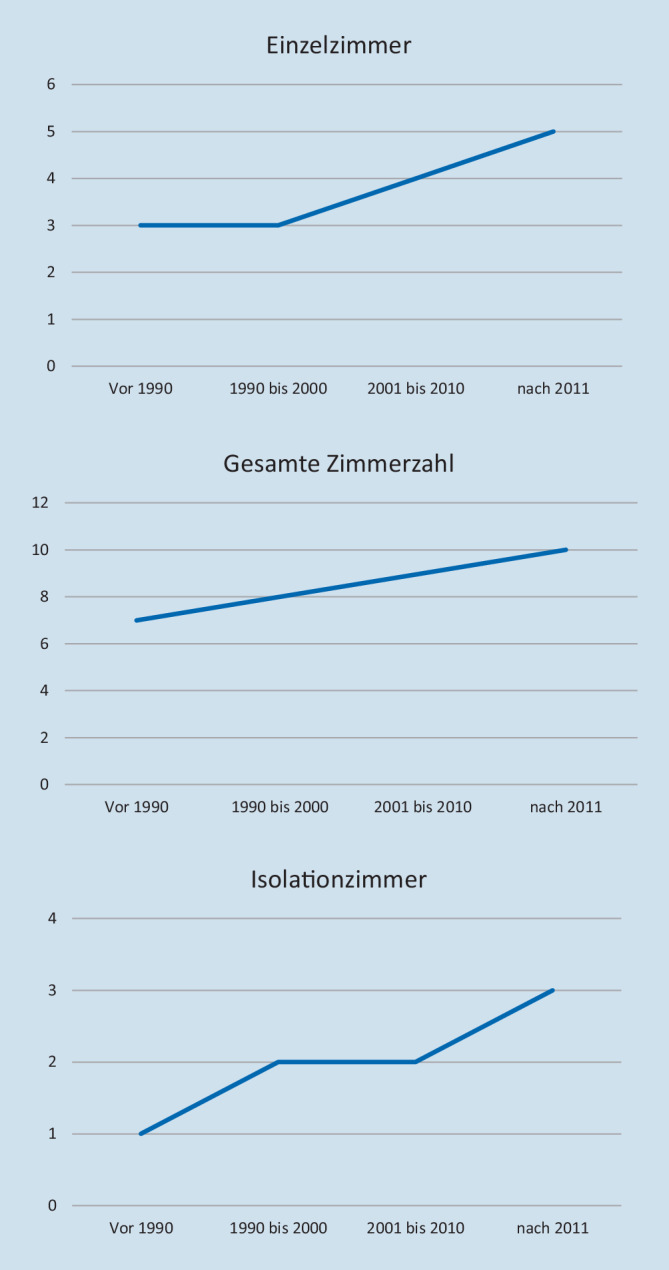

Es zeigen sich allerdings einige Trends über die Zeit (ITS gebaut vor 1990 vs. ITS gebaut nach 2011). Die Anzahl der Einbettzimmer (3 [IQR 2;5] vs. 5 [IQR 2;8]; *p* < 0,001) hat zugenommen. Parallel hat aber auch die Gesamtbettenanzahl zugenommen (7 [IQR 6;10] vs. 10 [IQR 8;14]; *p* < 0,001), sodass der Anteil an Einzelzimmer an der Gesamtzimmeranzahl der ITS sich nicht geändert hat (50 %; Abb. 1). Die Zahl der Vorräume (1 [IQR 0;2] vs. 2 [IQR 1;4]), die Zahl der Einbettzimmer mit Vorraum (1 [IQR 1;2] vs. 3 [IQR 2;4]; *p* < 0,001) und die Zahl der Vorräume, die mit Steckbeckenspülen ausgestattet sind (1 [QR 0;2] vs. 2 [IQR 1;4]), hat ebenfalls zugenommen (Abb. 1).

Neuere ITS haben im Schnitt auch mehr unreine Arbeitsräume (2 [IQR 1–2] vs. 1 [IQR 1–2]). Das ermöglicht häufiger eine feste Zuordnung zwischen Patientenzimmern und unreinen Räumen (30,8 % vs. 52,2 %).

Sowohl Einzelzimmer (17 [IQR 15–20] vs. 20 m^2^ [IQR 17,92–24]) als auch Mehrbettzimmer (27,5 [IQR 21,7–35,0] vs. 33 m^2^ [IQR 29,3–37,2]) sind tendenziell etwas größer geworden. Auch der Abstand zwischen den Patientenbetten (Seite zu Seite) hat leicht zugenommen (155 [IQR 132,5–200] vs. 200 cm [IQR 150–250]; *p* < 0,001).

ITS in Krankenhäusern mit höherem Versorgungslevel haben mehr Ein- und Mehrbettzimmer, mehr Vorräume und mehr Isolierzimmer. Hier findet man auch häufiger Patientenzimmer mit regulierbaren Luftdruckverhältnissen. Auch Krankenhäuser in öffentlicher Hand haben häufiger mindestens ein Isolierzimmer (69,2 % vs. 55,4 %; *p* = 0,034) und im Median auch mehr Einzelzimmer (4 [IQR 2–6] vs. 3 [IQR 1–5]; *p* = 0,001) und einen höheren Anteil an Einzelzimmern (46 % vs. 37 %; *p* = 0,003) im Vergleich mit privaten Krankenhäusern. Computer am Patientenbett und berührungslose Technik für Armaturen und Zimmertüren sind in Krankenhäusern der Maximalversorgung und Schwerpunktversorgung mehr verbreitet. Ein Computer für die Dokumentation im Patientenzimmer ist häufiger in öffentlichen als in privaten Krankenhäusern zu finden (48 % vs. 35 %).

Größere ITS (≥ 12 Betten), ITS in größeren Krankenhäusern (≥ 400 Betten) und ITS in Krankenhäusern mit höherem Versorgungslevel (Maximalversorgung, Schwerpunktversorgung) haben tendenziell mehr Lagerraumplatz, trotzdem müssen auf diesen ITS häufiger Geräte und Materialien in zusätzlichen Räumen gelagert werden.

In kleineren ITS wird das Wasser aus der Wasserleitung häufiger für die Patientenversorgung benutzt (89 % vs. 79 %; *p* = 0,02) sowie auf ITS in kleineren Krankenhäusern (89 % vs. 77 %; *p* = 0,003) und in Krankenhäusern der Grund- und Regelversorgung (87,6 % vs. 77,8 %; *p* = 0,01).

Größere ITS haben häufiger sowohl eine reine Arbeitsfläche als auch einen Medikamentenvorrat im Zimmer.

Eine komplette Auflistung der gefundenen signifikanten Unterschiede ist in Tab. 8 angegeben.

**Table 8:** 

Struktur der Intensivstation	Baujahr	Träger	Fachrichtung	Größe der ITS (</≥ 12 Betten)	Größe des Hauses (</≥ 400 Betten)	Versorgungslevel
Architektur der Intensivstation	–	–	–	–	–	–
Wann ist Ihre Intensivstation gebaut worden? (nach Jahrzehnten)	–	–	neu/wea am neuesten (kleine Zahlen)	≥ 12 Betten eher neuer	–	–
Gibt es auf der Intensivstation eine Schleuse für die Einschleusung des Personals?	–	Öffentlich mehr	neu/wea weniger (kleine Zahlen)	–	Mehr in KH < 400 Betten	–
Gibt es auf der Intensivstation eine Schleuse ausschließlich für die Einschleusung von Besuchenden?	–	–	–	Mehr in ITS ≥ 12 Betten	–	–
Gibt es auf der Intensivstation eine Schleuse ausschließlich für die Versorgung bzw. Entsorgung von Materialien?	–	–	–	Mehr in ≥ 12 Betten	–	–
Werden saubere Materialien als auch kontaminierte Materialien über dieselbe Schleuse ein- und ausgeschleust?	–	–	–	–	Mehr in KH < 400 Betten	–
Wie viel Platz (Gesamtfläche) ist als Lagerraum auf Ihrer Station deklariert (Angabe in m^2^)?	–	–	chir/nch/kch und iid > 400 am meisten	Mehr in ≥ 12 Betten	Mehr in KH ≥ 400 Betten	Mehr S/M
Werden Geräte/Materialien ausschließlich in dafür deklarierten Räumen gelagert?	–	–	–	Weniger in ≥ 12 Betten	Mehr in KH < 400 Betten	Mehr G/R
Findet die Aufbereitung von benutzten Patientenbetten auf der Station statt?	–	Privat mehr	wea/iid < 400 am häufigsten	–	Mehr in KH < 400 Betten	–
Wie viele Pflegestützpunkte gibt es auf Ihrer Intensivstation?	–	–	–	–	–	Mehr S/M
Befindet sich der Pflegestützpunkt in einem räumlich abgeschlossenen Bereich (dazu gilt auch Tresen mit Glasscheiben)? Bei mehr als einem Pflegestützpunkt bitte nur einen beschreiben	–	–	wea/neu am meisten	–	–	–
Wie viele Arbeitsplätze sind in dem Pflegestützpunkt vorhanden? Bei mehr als einem Pflegestützpunkt bitte nur einen beschreiben	Zunahme	–	–	Mehr ≥ 12 Betten	–	–
Befindet sich ein Arztzimmer auf Ihrer Intensivstation? (Kein OA-Büro gemeint)	–	–	wea am wenigsten	Mehr in ≥ 12 Betten	Mehr in KH ≥ 400 Betten	–
Wie groß ist dieses Arztzimmer (Angabe in m^2^)?	Zunahme	–	neu/nch am größten	Größer in ≥ 12 Betten	–	–
Findet in dem Arztzimmer eine Lüftung über RLT-Anlage statt?	–	–	neu (kleine Zahlen) am wenigsten	–	Mehr in KH ≥ 400 Betten	–
Verfügt dieses Arztzimmer über die Möglichkeit der Fensterlüftung?	–	–	–	–	Mehr in KH < 400 Betten	–
Wie viele Arbeitsplätze sind in dem Arztzimmer vorhanden?	Zunahme	–	iid ≥ 400 und ime am meisten	Mehr in ≥ 12 Betten	Mehr in KH ≥ 400 Betten	Mehr S/M
Gibt es auf Ihrer Intensivstation einen oder mehrere reine Arbeitsräume außerhalb der Patientenzimmer für die Vorbereitung von Infusionen oder für andere aseptische Tätigkeiten?	Zunahme	–	–	–	–	–
Wie viele (reine Räume)?	–	–	–	Mehr in ≥ 12 Betten	–	Mehr S/M
Wie viele (unreine Räume)?	Zunahme	–	–	Mehr in ≥ 12 Betten	Mehr in KH ≥ 400 Betten	Mehr S/M
Gibt es eine feste Zuordnung zwischen Patientenzimmern und unreinen Arbeitsräumen?	Zunahme	–	–	–	–	–
Gibt es auf Ihrer Intensivstation einen oder mehrere Aufenthaltsräume für das Personal?	–	–	wea am wenigsten	–	–	–
Nennen Sie bitte die kumulative Größe der Aufenthaltsräume? (Angaben in m^2^)	Zunahme	–	iid < 400 und paed am kleinsten	Mehr in ≥ 12 Betten	Mehr in KH ≥ 400 Betten	–
Findet in den Patientenzimmern eine Lüftung über RLT-Anlage statt?	–	–	wea/neu am wenigsten	–	Mehr in KH ≥ 400 Betten	Mehr S/M
Kann in mindestens einem Patientenzimmer durch die Lüftungsanlage ein Über- bzw. Unterdruck zur Schleuse/Flur generiert werden?	–	–	iid > 400, chi, paed am meisten, neu/wea am wenigsten	–	–	Mehr S/M
Verfügen die Patientenzimmer über die Möglichkeit der Fensterlüftung?	–	–	chir/nchi/kch am wenigsten	–	Mehr in KH < 400 Betten	Mehr G/R
Wie viele Vorräume/Schleusen sind insgesamt auf Ihre Intensivstation vorhanden?	Zunahme	–	kch/neu am wenigsten	Mehr in ≥ 12 Betten	–	Mehr S/M
Verfügen eines oder mehrere Einbettzimmer über einen eigenen Vorraum/Schleuse?	Zunahme	Öffentlich mehr	kch/neu am wenigsten	–	–	–
Wie viele Einbettzimmer verfügen über einen eigenen Vorraum/Schleuse?	Zunahme	–	kch am meisten	Mehr in ≥ 12 Betten	Mehr in KH ≥ 400 Betten	Mehr S/M
Wie viele von diesen Vorräumen/Schleusen sind mit Unterdruckoption ausgestattet?	–	–	–	–	–	Mehr S/M
Wie viele von diesen Vorräumen/Schleusen verfügen über einen unreinen Arbeitsbereich mit Steckbeckenspüle?	Zunahme	–	nchi/neu/paed am wenigsten	–	Mehr in KH ≥ 400 Betten	Mehr S/M
Wie viele von diesen Vorräumen/Schleusen verfügen über einen unreinen Arbeitsbereich mit Steckbeckenspüle?	Zunahme	–	–	–	–	–
Sind zimmerübergreifende Schleusen auf der Intensivstation vorhanden (eine Schleuse, aus der mehr als ein Patientenzimmer zugänglich ist)?	Zunahme	–	iid ≥ 400 am meisten	–	–	–
Wird UVC-Strahlung zur Desinfektion der Luft oder von Oberflächen eingesetzt?	–	–	–	–	–	Mehr S/M
Über wie viele Einbettzimmer verfügt Ihre Intensivstation?	Zunahme	Öffentlich mehr	paed/nch am wenigsten	–	Mehr in KH ≥ 400 Betten	Mehr S/M
Anteil Einzelzimmer an Zimmer gesamt	Zunahme	Öffentlich mehr	–	Mehr in < 12 Betten	–	–
Anzahl Zimmer gesamt	Zunahme	Öffentlich mehr	paed/nch am wenigsten	–	–	Mehr S/M
Wie groß ist das Zimmer? (Angaben in m^2^)	Zunahme	–	–	–	–	–
Befindet sich ein patientenbezogener Computer für die Eingabe von Anordnungen bzw. für die Dokumentation von Behandlungen im Patientenzimmer?	–	Öffentlich mehr	iid < 400 /neu am wenigsten	–	Mehr in KH ≥ 400 Betten	Mehr S/M
Befindet sich dieser Computer direkt am Patientenbett?	–	Öffentlich mehr	–	–	–	–
Wird das Wasser aus der Wasserleitung für die Patientenversorgung genutzt?	–	–	–	Mehr in < 12 Betten	Mehr in KH < 400 Betten	Mehr G/R
Ist das Waschbecken mit Filter ausgestattet?	Abnahme	–	neu/wea/chir am wenigsten	–	Mehr in KH ≥ 400 Betten	Mehr S/M
Können die Armaturen ohne Handkontakt bedient werden?	–	–	–	–	–	Mehr S/M
Ist im Patientenzimmer eine reine Arbeitsfläche vorhanden?	Zunahme	–	–	Mehr in ≥ 12 Betten	–	–
Ist im Patientenzimmer eine unreine Lagerfläche (für Bettzeug, Kissen etc.) vorhanden?	Zunahme	–	–	–	–	–
Ist ein Abwurf für Wäsche vorhanden?	–	–	iid <400 weniger	Mehr in ≥ 12 Betten	Mehr in KH ≥ 400 Betten	Mehr S/M
Werden im Patientenzimmer Medikamente als Vorrat gelagert?	–	–	–	Mehr in ≥ 12 Betten	Mehr in KH ≥ 400 Betten	Mehr S/M
Kann die Zimmertür ohne Handkontakt bedient werden (wenn das Zimmer ein Vorraum hat, bitte nur die Tür zum Zimmer angeben)?	–	–	–	–	Mehr in KH > 400 Betten	Mehr S/M
Über wie viele 2‑ oder Mehrbettzimmer (> 2-Betten) verfügt Ihre Intensivstation?	Zunahme	–	iid ≥ 400, chi/kch am meisten	Mehr in ≥ 12 Betten	Mehr in KH ≥ 400 Betten	Mehr S/M
Wie groß ist das Zimmer (Angaben in m^2^)?	Zunahme	–	–	Mehr in ≥ 12 Betten	Mehr in KH ≥ 400 Betten	–
Wie groß ist der Abstand zwischen den Betten von (Angaben in cm)?	Zunahme	–	–	–	–	Mehr S/M
Ist eine Abtrennung zwischen den Betten vorhanden?	–	–	paed weniger	–	Mehr in KH < 400 Betten	–

*Baujahr:* Vor 1990/ab 2011, *Träger* öffentlich/privat, *Fachrichtung:* iid<400 – interdisziplinäre ITS im Krankenhaus mit <400 Betten, iid>400 – interdisziplinäre ITS im Krankenhaus mit ≥400 Betten, *ime* internistisch, *chir* chirurgisch, *nch* neurochirurgisch, *kch* kardiochirurgisch, *neu* neurologisch, *paed* paediatrisch, *wea* weaning, *Versorgungslevel*: S/M - Schwerpunkt und Maximalversorger (inkl. Universität), G/R - Grund-, Regelversorgung/Fachkrankenhaus/Reha.

^a^Unterschiede wurden mittels χ^2^ oder Wilcoxon Rangsummentest auf Signifikanz geprüft. Signifikant war ein *p*-Wert < 0,05

## Diskussion

### Einbettzimmer

Bereits 1995 hat die Kommission für Krankenhaushygiene und Infektionsprävention (KRINKO) empfohlen, in Intensivbereichen vorwiegend Einzelzimmer bei Neubauten zu planen. Mehrere Studien mit einem „Before-after-Design“ haben eine Reduktion des Erwerbs von Infektionen auf ITS nach einem Wechsel von einer Station mit überwiegend Mehrbettzimmern zu einer alleinigen Einzelzimmerstruktur, nachgewiesen [7, 8, 16, 21]. Dieser Trend wird bereits in Europa, v. a. im skandinavischen Raum, und in den USA umgesetzt und in der aktuellen Fassung der Facility Guidelines for Institutions (FGI; https://fgireadonly.madcad.com/) für die USA wird z. B. generell empfohlen, ITS ausschließlich mit Einzelzimmern zu planen.

Die Deutsche Gesellschaft für Krankenhaushygiene (DGKH) hat in einem Positionspapier 2021 die aktuelle Evidenz zu Vor- und Nachteilen der Unterbringung in Einzelzimmern zusammengefasst und schließlich für den Neubau von ITS einen mindestens 70 %igen Einzelzimmeranteil empfohlen. Die DIVI hat diese Mindestforderung in ihren aktuellen Empfehlungen übernommen [11, 23].

In unserer Umfrage scheint sich ein Trend zu mehr Isolierräumen und etwas größeren Zimmern durchzusetzen. Diese Entwicklung vollzieht sich allerdings sehr langsam und immer noch sind ca. 65 % der Intensivbetten in Mehrbettzimmern aufgestellt. Vor allem in privaten Krankenhäusern und in größeren ITS ist der Anteil an Betten in Einzelzimmern noch viel zu klein. Das kann bei der aktuellen Zunahme an isolierpflichtigen Patienten oder solchen mit multiresistenten Erregern – wie durch die COVID-19-Pandemie eindeutig sichtbar gemacht wurde – zu einem kritischen Engpass für die Versorgung führen. Darüber hinaus liegt die Durchschnittsgröße der Zimmer noch unter den von der European Society of Intensive Care Medicine (ESICM) und der Deutschen interdisziplinären Vereinigung für Intensivmedizin (DIVI) empfohlenen Richtwerten von 25 m^2^ für Einzelzimmer und 40 m^2^ für Doppelzimmer [23].

### Wasserversorgung und Händehygiene

Unsere Daten zeigen, dass Waschbecken in Patientenzimmern noch der Standard sind. Vor allem auf kleinen ITS, auf ITS in kleinen Krankenhäusern und in Krankenhäusern der Grund- und Regelversorgung wird regelmäßig Wasser aus der Leitung für die Patientenversorgung benutzt. Die Assoziation zwischen kontaminierten Wasserstellen und nosokomialen Infektionen ist allerdings mittlerweile gut belegt [14] und die KRINKO empfiehlt deshalb seit 2018 bei Neubauten auf den Einbau von Waschbecken im Patientenzimmer – v. a. in Bereichen mit infektionsgefährdeten Patienten – zu verzichten [2]. Zukünftig sollte eine Balance zwischen Funktionalität des Zimmers (z. B. schneller Zugang zum Wasser für das Pflegepersonal) und Patientensicherheit gefunden werden. Die Verwendung von Wasserfiltern ist in den neueren ITS und in ITS in kleineren Krankenhäusern weniger häufig. Ob das mit einer geringeren Kontamination des Wassersystem mit Legionellen zusammenhängt, kann anhand dieser Daten nicht gesagt werden.

Darüber hinaus empfiehlt die WHO schon seit 2009, einen Händedesinfektionsmittelspender in Armlänge vom Patientenbett zu positionieren, um die Compliance zur Händedesinfektion zu erhöhen [3]. Das scheint in den meisten ITS auch so zu sein (75,4 % am Patientenbett). In Anbetracht der guten Evidenz dafür bleibt allerdings schwer erklärbar, dass von einem Viertel der Stationen diese Empfehlung noch nicht umgesetzt wird.

### Lüftung

In dieser Studie verfügen 86,4 % der Patientenzimmer, 80,2 % der Stützpunkte und 55,7 % der Arztzimmer über eine RLT-Anlage, die einen kontinuierlichen Luftaustausch gewährleistet. Fensterlüftung ist in 76,5 %, 31 % und 83,5 % der oben genannten Räume möglich. Aus unserer Umfrage geht nicht hervor, wie die konkrete Leistung der jeweiligen RLT-Anlagen ist und ob eine Luftfilterung angewendet wird. ITS in größeren Krankenhäusern und in Krankenhäusern eines höheren Versorgungslevels sind in Bezug auf RLT-Anlagen besser ausgestattet. Die Rolle der Raumbelüftung in der Prävention von luftübertragbaren Infektionen auf der ITS wird in der Literatur allerdings seit einiger Zeit diskutiert [17, 19] und Outbreak-Berichte im Rahmen der COVID-19-Pandemie identifizierten nicht gut konzipierte Belüftungssysteme als Risikofaktor [1, 10]. Für die zukünftige Planung und Sanierung von ITS ist es aus unserer Sicht sinnvoll, dem Thema Lüftungssysteme einen höheren Stellenwert beizumessen.

### Sonstige Aspekte

Obwohl die Mindestanzahl an geforderten Funktionsräumen (reine/unreine Räume, Lagerräume usw.) in den meisten ITS eingehalten wird, bleibt unklar, inwiefern diese auch dem tatsächlichen Bedarf gerecht werden. Die als solche zur Verfügung stehenden Lagerräume sind in ihrer Fläche häufig unzureichend groß (55 % der Stationen müssen Materialien anderswo unterbringen). Interessanterweise zeigt sich, dass, obwohl größere ITS und ITS in größeren Krankenhäusern mehr Lagerplatz haben, genau auf diesen Stationen häufiger Materialien in sonstigen Räumen gelagert werden. Das lässt vermuten, dass die Planung von Lagerräumen zu knapp ist. Eine dezentrale Aufbereitung von Betten und Geräten findet häufiger auf ITS in kleineren und in privaten Krankenhäusern statt. Für diese Prozesse gibt es aber oft keine zugewiesenen Räume. Insgesamt 66 % der ITS haben keinen Raum für die Aufbereitung von Geräten, 60,4 % bereiten ihre Betten im Patientenzimmer auf und 35,5 % haben keine definierten separaten Abstellplätze für saubere bzw. unsaubere Betten. Inadäquate Aufbereitung von „high-touch surfaces“ führt zu mehr Umgebungskontamination [9] und kontaminierte Gegenstände sind als Quelle für Infektionen identifiziert worden [12]. Deshalb ist eine zentrale Aufbereitung mittels standardisierter Verfahren im Allgemeinen zu präferieren. Für die zukünftige Planung ist es aber wichtig, die notwendige Dezentralisierung von einigen Prozessen zu berücksichtigen, damit diese unter den besten Bedingungen erfolgen können.

Die vorgestellte Untersuchung hat die Stärke, dass eine große Anzahl von ITS teilgenommen hat. Dabei wurden ITS unterschiedlicher Größe, Fachrichtung und Versorgungslevel einbezogen. Das legt nahe, dass die Ergebnisse einen guten Überblick über die Realität in Deutschland geben. Anhand der Grundcharakteristika der teilnehmenden ITS kann eine Selektion in eine bestimmte Richtung weitgehend ausgeschlossen werden.

Die Studie hat auch Limitationen. Lediglich 39,8 % der eingeschlossenen ITS haben an der Umfrage teilgenommen. Darüber hinaus wurden zur Begrenzung der Arbeitslast die Teilnehmer gebeten, ein einziges, möglichst „typisches“ Einbett- und Mehrbettzimmer zu beschreiben. Aus unseren Erfahrungen haben allerdings meistens alle Zimmer auf einer ITS eines Krankenhauses eine ähnliche Ausstattung, sodass es aus unserer Sicht dadurch zu keinem großen Informationsverlust gekommen sein sollte.

## Fazit für die Praxis

- In Deutschland zeigt sich ein Trend zu mehr Einzelzimmern und mehr Isolationsmöglichkeiten, dieser Trend vollzieht sich allerdings sehr langsam.
- Aus infektionspräventiver Sicht gibt es in vielen Strukturen einen dringlichen Sanierungsbedarf. Vor allem ITS in privaten Krankenhäusern und in Krankenhäusern mit niedrigerem Versorgungslevel weisen größere Lücken in der Ausstattung auf, um geltenden Anforderungen in Bezug auf z. B. Einzelzimmerausstattung und Lüftungssysteme gerecht zu werden.
- 20 % der befragten ITS in Deutschland wurden vor 1990 gebaut. Ältere bauliche Strukturen können nur mit größerem Aufwand an die geltenden Anforderungen/Empfehlungen angepasst werden. In einigen Fällen könnte der Neubau nach modernen Konzepten die bessere Alternative darstellen.
- Das Anpassen der vorhandenen ITS an neue zukunftsweisende Konzepte wird aufwendige Sanierungen und den Neubau von weiteren Strukturen erfordern. Die entsprechende Finanzierung muss in der Krankenhausplanung berücksichtigt werden.
